# Liddle syndrome presenting with normal aldosterone levels: A case report

**DOI:** 10.1097/MD.0000000000035944

**Published:** 2023-11-24

**Authors:** Rongrong Wang, Yan Zhang, Runzhou Pan, Rongju Zhang, Yongcai Zhao

**Affiliations:** a Cangzhou Central Hospital, Cangzhou, Hebei, China.

**Keywords:** hypertension, hypokalemia, Liddle syndrome, primary aldosteronism

## Abstract

**Introduction:**

Liddle syndrome is an autosomal dominant disorder characterized by hypertension, hypokalemia, low aldosterone levels, and reduced renin activity. Atypical Liddle syndrome can be easily misdiagnosed due to its clinical phenotypes resembling hyperaldosteronism.

**Patient concern:**

The patient was diagnosed with primary aldosteronism due to hypertension and hypokalemia, and underwent left adrenalectomy. After the operation, the patient still had hypertension and hypokalemia that were not easy to control and correct, and had acute cerebral infarction.

**Diagnosis:**

The genetic test showed that the base duplication in the coding region of SCN1B gene caused a frameshift mutation:c.1789dupC (p.Arg597fs), Liddle syndrome was diagnosed.

**Intervention and outcomes:**

The patient was treated with a low-sodium diet and oral triamterene. The serum potassium level returned to normal and the blood pressure was controlled.

**Lessons:**

Some Liddle syndrome may present with normal aldosterone levels, genetic testing is necessary for the diagnosis. If the diagnostic test of primary aldosteronism is positive, but the treatment with spironolactone is ineffective, we should actively search for other causes.

## 1. Introduction

Liddle syndrome is an autosomal dominant hereditary disease characterized by early onset, uncontrolled hypertension, hypokalemia, low aldosterone, and renin activity levels, with or without metabolic alkalosis. It often presents with a family history of hypertension and can also occur sporadically. Aldosterone receptor antagonists are ineffective in treating Liddle syndrome; however, epithelial sodium channel blockers have been found to be effective. We report a case with clinical manifestations similar to primary aldosteronism (PA), but Liddle was confirmed by gene, so as to strengthen the understanding of Liddle and avoid misdiagnosis.

## 2. Case presentation

**Figure 1.**
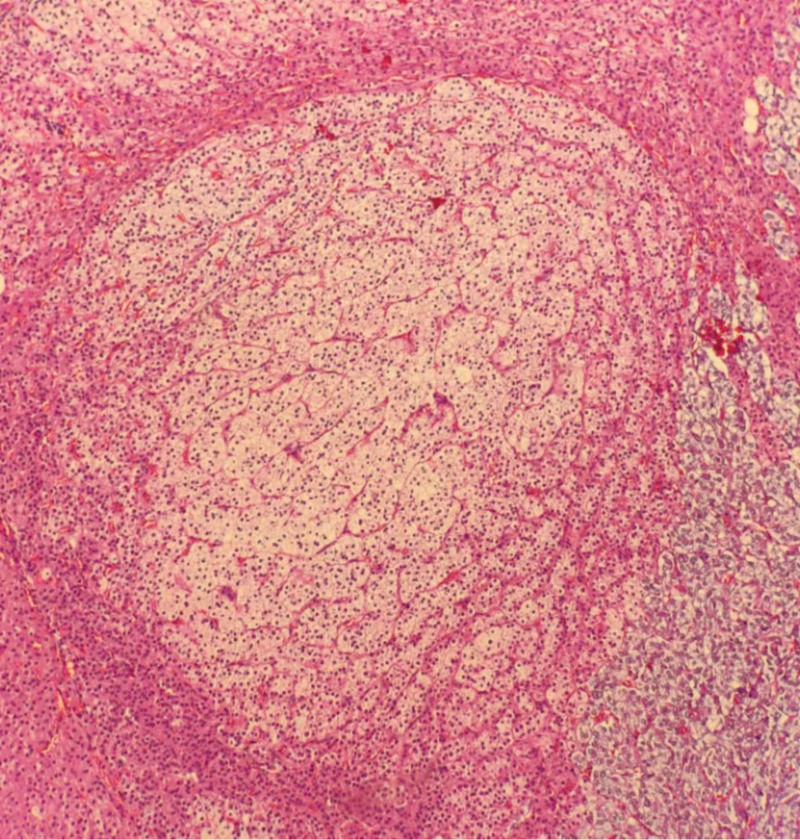
Contrast-enhanced CT of the adrenal gland: The left adrenal gland was slightly thickened with homogeneous enhancement.

A 30-year-old woman was admitted to the hospital with a complaint of elevated blood pressure for 13 years and slurriness of speech for 3 days. Hypertension had been noted 13 years earlier and hypokalemia 10 years earlier. Three years ago, supine and standing aldosterone: (supine): plasma aldosterone concentration (PAC):180.742 pg/mL (reference range:30–300), plasma renin activity (PRA):0.067 ng/mL/h (reference range: 0.1–6.56); (upright position):

PAC:178.443 pg/mL, PRA:0.025 ng/mL/h. Captopril test: (before captopril) PAC:157.684 pg/mL, PRA:0.26 ng/mL/h; (2 h after captopril): PAC141.442 pg/mL, PRA 1.17 ng/mL/h. Enhanced CT of the adrenal gland (Fig. 1):the left adrenal gland was slightly enlarged, considering nodular hyperplasia?. Multiple cysts were observed in both kidneys.

**Figure 2.**
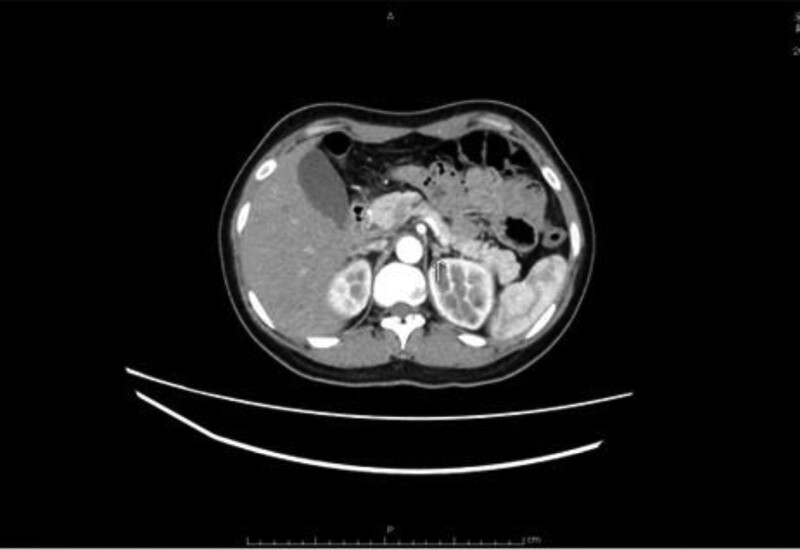
Pathology after left adrenalectomy: nodular hyperplasia of the adrenal cortex.

The patient was diagnosed with primary aldosteronism, but had a poor response to oral spironolactone. One month later, the patient underwent left adrenalectomy. Pathology showed nodular hyperplasia of the adrenal cortex (Fig. 2). The patient did not take antihypertensive and potassium supplement drugs after discharge. Three days before admission, she had slurred speech and fatigue. Her blood potassium was 2.8 mmol/L, and she was admitted for treatment. The physical examination revealed a blood pressure reading of 155/108 mm Hg, clear consciousness, and no discernible abnormalities in the heart, lungs and abdomen. Tanner: 5. Family history: Her mother died of cerebral hemorrhage at the age of 40. Her father died of an accident.

**Figure 3.**
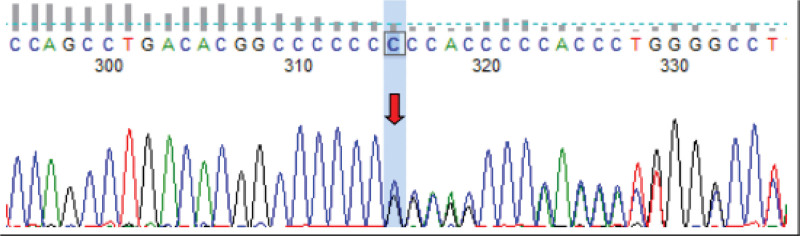
The nucleotide repeat cytosine at position 1789 in the coding region of the SCN1B gene results in a frameshift mutation following the amino acid arginine at position 597.

Reevaluated after surgery: PAC 26.31 pg/mL (reference range: 70–300), PRA 0.39 ng/mL/h (reference range: 0.1–6.56). (8:00) cortisol 10.91 µg/dL; (16:00) cortisol 4.460 µg/dL; (0:00) cortisol: 1.620 µg/dL. Twenty-four hours urine free cortisol:39.2 µg/24 h (reference range: 26–127.55); blood gas analysis showed PH 7.46, BE 3.5, HCO3-C 27.6. ACTH, 17-hydroxyprogesterone and sex hormones were normal, and bilateral renal artery ultrasound showed no obvious abnormalities. CT imaging of the adrenal gland revealed absence of left adrenal gland visualization and no abnormalities in the right adrenal gland. Brain magnetic resonance imaging showed acute cerebral infarction on the right side of pons. Genetic testing showed a frameshift mutation of c.1789dupC (p.Arg597fs) caused by base duplication in the coding region of the SCN1B gene (Fig. 3), which was consistent with Liddle syndrome. The patient was given a low-salt diet and oral triamterene 50 mg/d. After 2 weeks of medication, the blood potassium was 4.48 mmol/L (reference range: 3.5–5.3), the blood sodium was 147.5 mmol/L (137–147), and the blood pressure was 125/85 mm Hg. Due to the high level of serum sodium, the dose of triamterene was reduced to 37.5 mg/d orally. The blood potassium and blood pressure were well controlled.

## 3. Discussion

Liddle syndrome was initially described in 1963, and its pathogenesis is attributed to mutations in the ENaC gene located in the distal renal tubular epithelium. ENaC is an amiloride sensitive epithelial sodium channel composed of 3 homologous subunits, α, β and γ, encoded by the SCNN1A, SCNN1B, and SCNN1G genes, respectively. Each subunit has a highly conserved sequence at its carboxyl terminal, known as the PY motif, which binds to the ubiquitin ligase Nedd4-2 and causes the internalization of ENaC and degradation.^[1]^ When the gene is mutated, the internalization and degradation of ENaC is dysfunctional, causing excessive Na + reabsorption, resulting in increased plasma volume and causing hypertension. Increased sodium ion flow in the apical membrane of distal renal tubular epithelial cells causes potassium and hydrogen ions to be secreted into the collecting duct, leading to hypokalemia and metabolic alkalosis.^[2]^ Hypertension and hypokalemia can inhibit the RASS system, which is manifested as low renin and aldosterone.

The patient had hypertension, hypokalemia, and PAC > 15 ng/mL, aldosterone to renin ratio (ARR) > 30, with a positive captopril test and a combination of unilateral adrenal enlargement, she was diagnosed PA. Left adrenalectomy was performed due to the ineffectiveness of oral spironolactone. Her PAC decreased after surgery, but still had hypokalemia and hypertension. During hospitalization, we ruled out congenital adrenal hyperplasia, Cushing syndrome, renal tubular acidosis, and renal-artery stenosis. Invite the superior hospital experts to consult: recommend genetic testing. After obtaining the patient’s informed consent, genetic testing was performed, and the result showed that the base duplication in the coding region of the SCN1B gene caused a frameshift mutation: c.1789dupC (p.Arg597fs), which was consistent with the diagnosis of Liddle syndrome. The gene mutation site of our case has been reported in the literature.^[3,4]^ Martina Tetti^[5]^ et al found that 58.2% of Liddle patients had decreased aldosterone. Liddle can present with normal aldosterone levels. plasma renin and aldosterone suppression is not only affected by gene mutations, other genetic and/or environmental factors, such as genes controlling sodium reabsorption or low salt intake, may affect Liddle phenotype.^[6]^ The ARR ratio is affected by many factors, and the diagnostic specificity of the increased ratio is not 100%.^[7]^ In the captopril trial, a suppression percentage of 30% as the cutoff caused many hypertensive patients to be misdiagnosed.^[8]^ In the 2020 Endocrine Hypertension consensus, 2 hours after captopril: PAC > 11 ng/dL and PRA remaining suppressed Or ARR > 20 (ng/dL)/ [ng/mL/h]: PA confirmed.^[9]^ Hypokalemia should be corrected before the test and the interference of some antihypertensive drugs should be excluded to avoid false positive or false negative results.^[10]^ If unilateral PA is considered, it can usually be confirmed by sampling the adrenal veins bilaterally rather than blind surgery.

For the treatment of Liddle syndrome, potassium-sparing diuretics such as amiloride and triamterene can reduce ENaC activity, and in combination with a sodium reduction diet can restore normal blood pressure and electrolyte imbalance in Liddle syndrome patients and animal models.^[11]^

The treatment sensitivity of ENac blockers in the same family is also different,^[6]^ so the drug dose should be adjusted in time according to blood pressure and ions during the treatment. The blood pressure and serum potassium of our patient were well controlled after the treatment with triamterene 50 mg/day. Because of the increase of serum sodium, the blood sodium returned to normal after reducing the dose of triamterene.

In conclusion, If the diagnostic test of PA is positive, but the treatment with spironolactone is ineffective, we should actively search for other causes. Aldosterone levels in Liddle may be normal, and genetic testing should be performed when necessary to avoid misdiagnosis, reduce unnecessary surgical trauma, and prevent the occurrence and development of serious complications such as cardiovascular and cerebrovascular diseases.

## Author contributions

**Conceptualization:** Rongrong Wang, Runzhou Pan.

**Data curation:** Yan Zhang, Rongju Zhang.

**Writing – original draft:** Rongrong Wang.

**Writing – review & editing:** Yongcai Zhao
